# Supplementary material for: Correction of Clcn1 alternative splicing reverses muscle fiber type transition in mice with myotonic dystrophy
Source: Nat Commun. 2023 Apr 7;14:1956. doi: 10.1038/s41467-023-37619-1 (PMC10082032; doi:10.1038/s41467-023-37619-1)
Supplement: Supplementary file 1 — Supplementary Information [file 41467_2023_37619_MOESM1_ESM.pdf]

Correction of Clcn1 alternative splicing reverses muscle fiber type transition in mice with myotonic dystrophy

Ningyan Hu, Eunjoo Kim, Layal Antoury, and Thurman M. Wheeler*

Department of Neurology, Massachusetts General Hospital and Harvard Medical School, Boston, MA, USA

*Corresponding author:

twheeler1@mgh.harvard.edu

CNY 149 - 6323

149 13th St.

Boston, MA 02129, USA

This file contains:

Supplementary Figs. 1 - 4

Supplementary Tables 1 - 3

Supplementary References

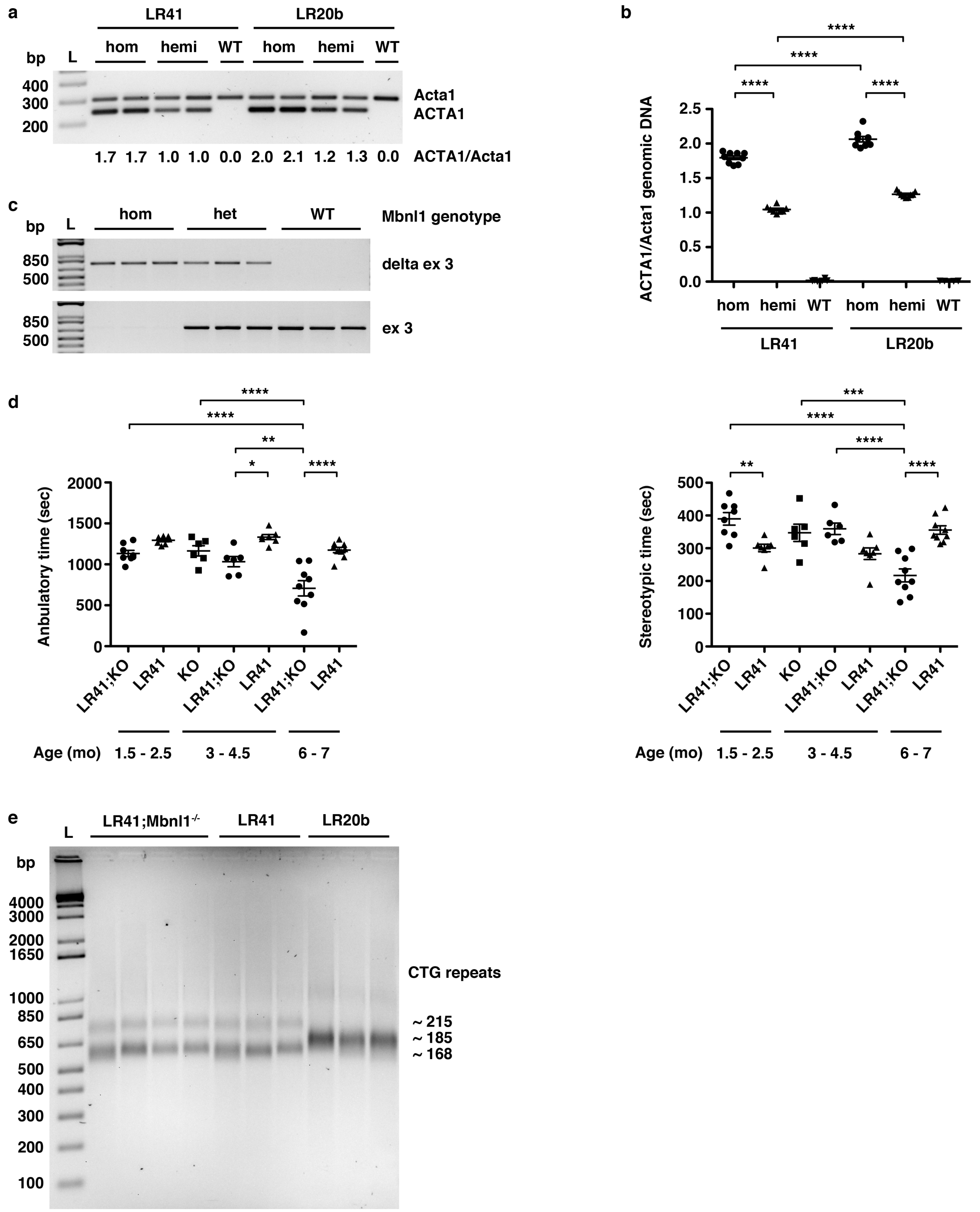

Supplementary Fig. 1 | Genotyping, progressive functional impairment, and CTG repeat length in the LR41;*Mbnl1*$^{-/-}$ double homozygous mouse model of DM1. We crossed HSA^{LR} line 41 transgenic[1] (LR41) mice with muscleblind-like-1 knockout[2] (*Mbnl1*$^{-/-}$) mice to create the LR41;*Mbnl1*$^{-/-}$ double homozygous model of DM1. a) PCR of tail biopsy genomic DNA using primers targeting the human *ACTA1* transgene and endogenous mouse *Acta1* enables determination of LR41 or LR20b transgene zygosity. A representative gel identifies homozygous (hom), hemizygous (hemi), or wild type (WT) mice based on the ratio of *ACTA1*/*Acta1* using band densitometry. b) Quantification of zygosity in the LR41 and LR20b lines. N = 9 homozygous, N = 8 or 9 hemizygous, and N = 5 or 6 WT each. **** $P < 0.0001$

(one-way ANOVA). Error bars indicate ± s.e.m. c) *Mbnl1*$^{-/-}$ mice have a homozygous deletion of *Mbnl1* exon 3[2]. Shown is PCR identification of zygosity for the *Mbnl1* exon 3 deletion using a common left primer and separate right primers specific for the exon 3 deletion (delta ex 3) or the wild type exon 3 (ex 3) in separate PCR reactions. Presence of exclusively the delta ex 3 allele defines homozygous knockouts (N = 3), presence of both the delta ex 3 and wild type ex 3 alleles defines heterozygotes (N = 3), and presence of exclusively the ex 3 allele defines wild types (N = 3). bp = base pairs; L = 1 Kb Plus DNA ladder. d) Quantification of spontaneous activity in LR41;*Mbnl1*$^{-/-}$ (LR41;KO), *Mbnl1*$^{-/-}$ (KO), and LR41 littermates as time (seconds; sec) spent walking (ambulatory; left) or on small rapid non-ambulatory movements such as scratching or grooming (stereotypic; right). We examined mice at ages 1.5 - 2.5 months, 3.5 - 4.5 months, and 6 - 7 months. The average of values for three separate 30-minute monitoring sessions for each individual mouse is shown. See Fig. 1. **** $P < 0.0001$, *** $P < 0.001$; ** $P < 0.01$; * $P < 0.05$ (one-way ANOVA). Error bars indicate ± s.e.m. e) PCR using 10 ng template genomic DNA and primers targeting the CTG repeat in the *ACTA1* transgene in LR41;*Mbnl1*$^{-/-}$ (LR41;KO), LR41, and LR20b (N = 3 each), followed by gel electrophoresis and staining with SYBR green. The uncropped gel is shown. CTG repeat length is estimated by subtracting the number of nucleotides amplified by the primers upstream and downstream of the CTG repeat from the total amplicon size and dividing by three[3]. The LR41 upper band = ~ 750 bp and the lower band = ~ 575 - 600. Therefore, CTG repeat length of LR 41 upper band = (750 - 95)/3 = 645/3 = approximately 215 and CTG repeat length of LR41 lower band = (575 - 95)/3 = 480/3 = approximately 160. Source data are provided as a Source Data file.

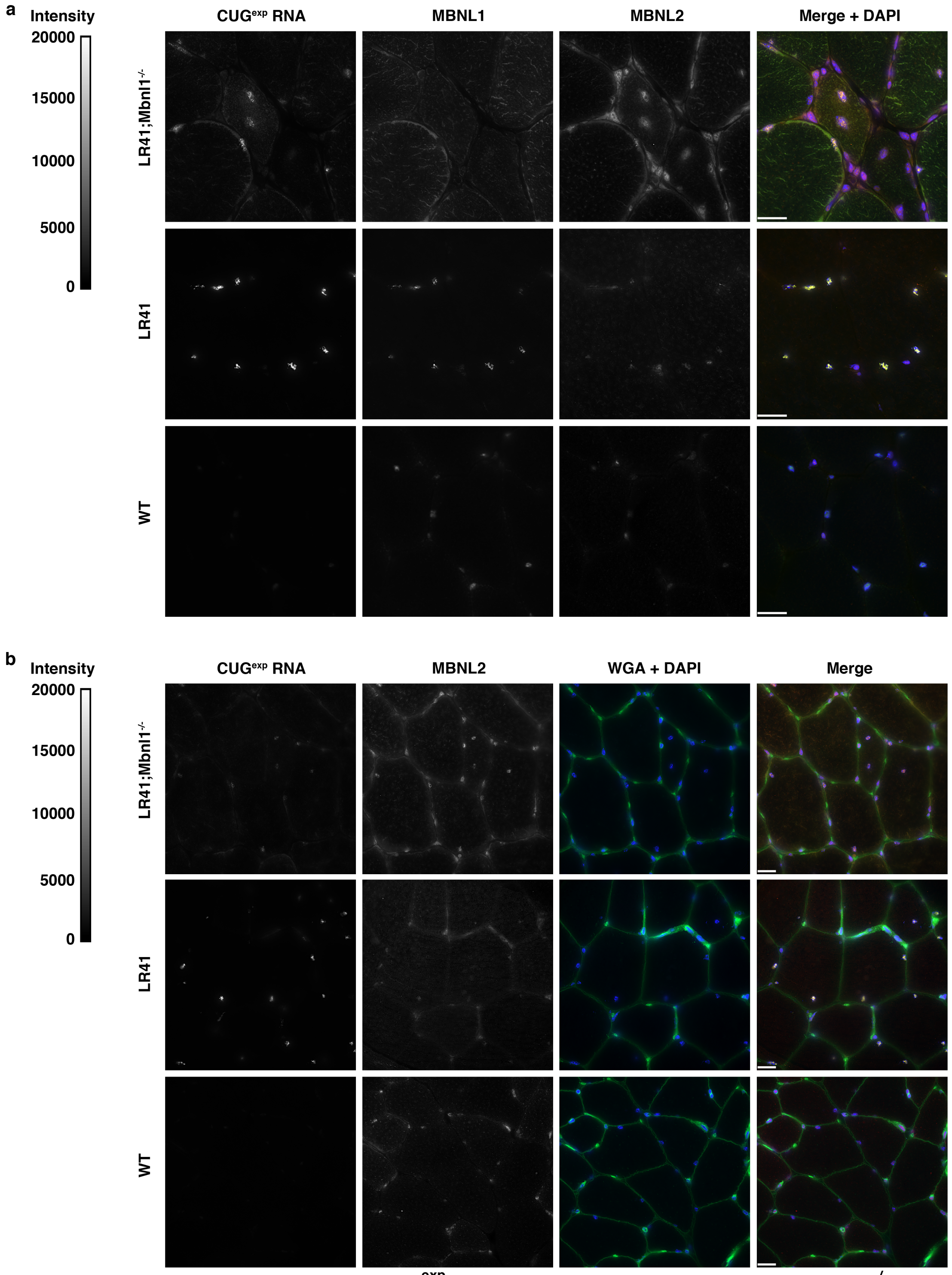

Supplementary Fig. 2 | Localization of CUG^{exp} RNA and MBNL2 protein in the LR41;*Mbnl1*$^{-/-}$ double homozygous mouse model of DM1. We used fluorescence *in situ* hybridization and immunofluorescence to localize *ACTA1*-CUG^{exp} RNA, MBNL1 protein, and MBNL2 protein in quadriceps muscle tissue of LR41;*Mbnl1*$^{-/-}$, LR41, and wild type (WT) (N = 4 each). a) Representative images of *ACTA1*-CUG^{exp} RNA, MBNL1 and MBNL2 protein. Fluorescence intensity is 0 - 20,000 gray scale units. In the merge images, *ACTA1*-CUG^{exp} RNA is yellow, MBNL1 green, and MBNL2 red. DAPI (blue) highlights nuclei. Bars indicate 20 μm. b) Representative images of *ACTA1*-CUG^{exp} RNA and MBNL2 protein. Fluorescence intensity is 0 - 20,000 gray scale units. In the merge images, *ACTA1*-CUG^{exp} RNA is yellow and MBNL2 red. Wheat germ agglutinin (WGA; green) outlines muscle fiber membranes and DAPI (blue) highlights nuclei. Bars indicate 20 μm.

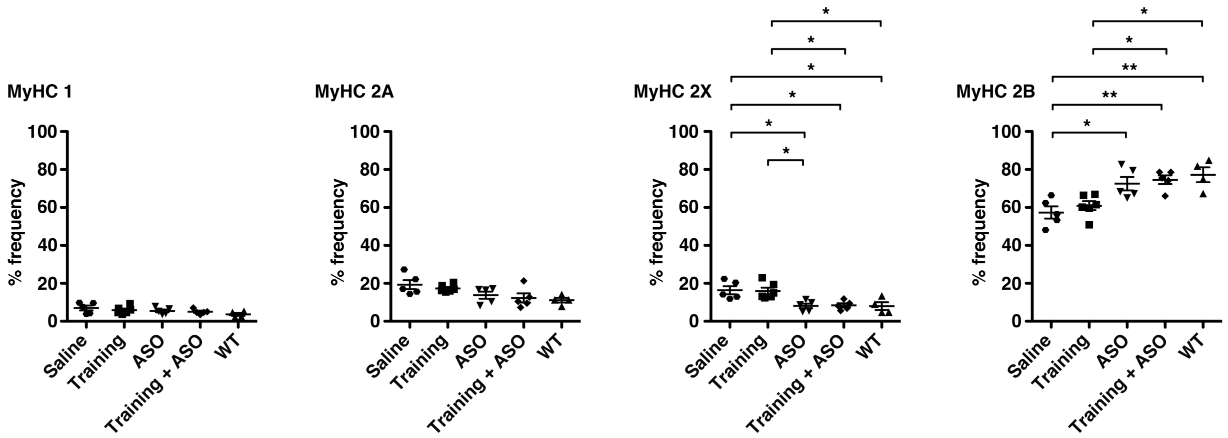

Supplementary Fig. 3 | Myosin heavy chain protein expression in old LR20b mice. Immunofluorescence quantification of myosin heavy chain Type 1 (MyHC 1), 2A (MyHC 2A), 2X (MyHC 2X), and 2B (MyHC 2B) protein expression in gastrocnemius muscles of 18 month-old LR20b treated with either saline, a treadmill walking exercise training regimen (Training), a systemic antisense oligonucleotide (ASO) targeting *ACTA1*-CUG^{exp} transcripts, or the training regimen combined with systemic ASO treatment (Training + ASO) (N = 5 or 6 each group), all for 3 ½ months duration beginning at ~ 14 ½ months of age[3]. Untreated age-matched wild type (WT) mice (N = 4) served as controls. ** $P < 0.01$; * $P < 0.05$ (one-way ANOVA). Error bars indicate ± s.e.m. Source data are provided as a Source Data file.

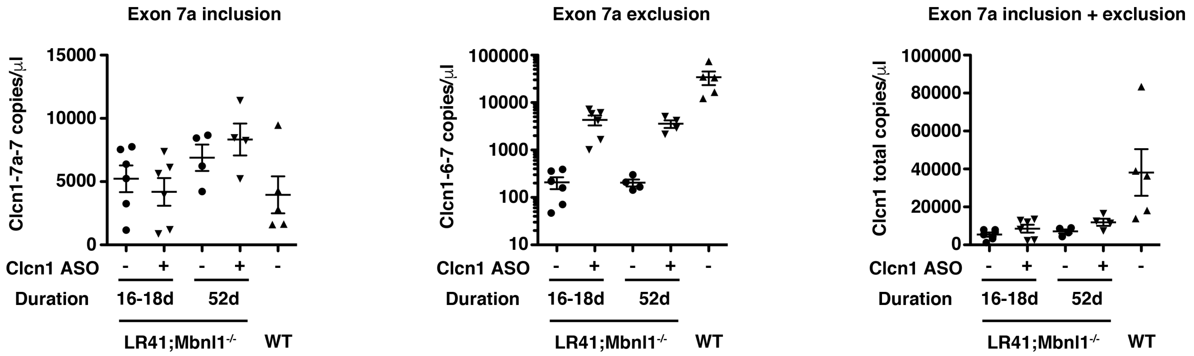

Supplementary Fig. 4 | ddPCR quantification of *Clcn1* transcripts. We injected TA muscles of LR41;*Mbnl1*$^{-/-}$ mice with Clcn1 ASO (+) designed to induce skipping, or exclusion, of *Clcn1* exon 7a. Contralateral muscles were treated with an oligo with a sequence that is a 5′-3′ invert of the Clcn1 ASO and has no target (-). Treatment duration was 16-18 (N = 6) or 52 days (N = 4). Untreated wild type (WT; N = 5) served as controls. *Clcn1* splice products were quantified as copies/*µ*l cDNA using primer probe sets specific for exon 7a inclusion (left), exon 7a exclusion (middle), and combined total of exon 7a inclusion + exclusion (right). These data were used to calculate % exon 7a inclusion (see Fig. 2). Error bars indicate ± s.e.m. Source data are provided as a Source Data file.

Supplementary Table 1 | PCR primers used to determine zygosity of *ACTA1* or *Mbnl1*, or estimate *ACTA1*-CTG^{exp} repeat length. The *Mbnl1* left primer and the right primer targeting *Mbnl1* delta exon 3 were designed using Primer3 software[4, 5]. Left and right primers for *ACTA1*, *Acta1*, and *ACTA1*-CTG, and the right primer for *Mbnl1* wild type exon 3 were published previously[2, 3, 6]. The amplicon size in base pairs (bp) is indicated for each.

Gene	Left primer (5′ - 3′)	Right primer (5′ - 3′)	Target	Amplicon size (bp)
ACTA1	AAACTTACATCTTCCCATGCTCC	GAGACGCCCTCTGAGAAACAG	*ACTA1* zygosity	249
Acta1	TCCTCAGGACGACAATCGAC	CCTAAGGAGTTCACCCAGTCTG	*Acta1* zygosity	310
ACTA1-CTG	TGCTGCCATCGTAAACTGAC	CTTCCACAGGGCTTTGTTTC	CTG expansion	varies
Mbnl1	TGGCTGCAATATGCCTCAC	TCTGTAAGCAGGACGATTGC	delta exon 3	777
Mbnl1	TGGCTGCAATATGCCTCAC	AGACCCTTTGACACCGAATTTC	wild type exon 3	681

Supplementary Table 2 | RT-PCR primers used to measure alternative splicing. Left and right primers for *Apt2a1*, *Cacna1s*, *Clasp1*, *Clcn1*, and *Ttn* were published previously[7-10]. The PCR product size in base pairs (bp) with exon inclusion (+ ex) or exon exclusion (- ex) is indicated for each.

Gene	Left primer (5′ - 3′)	Right primer (5′ - 3′)	Target exon	+ ex size (bp)	- ex size (bp)
Atp2a1	GCTCATGGTCCTCAAGATCTCAC	GGGTCAGTGCCTCAGCTTTG	22	218	176
Cacna1s	CCAGAGCTGCCTCTTCAAAATCG	GGAAGAAGGTGATGGAGATGCG	29	244	187
Clasp1	GTCGACGACAGGATCTCTCC	GAGCTCTGCCGTCTCGTG	20	222	174
Clcn1	TGAAGGAATACCTCACACTCAAGG	CACGGAACACAAAGGCACTG	7a	424	345
Titin	GTGTGAGTCGCTCCAGAAACG	CCACCACAGGACCATGTTATTTC	346	556	253

Supplementary Table 3 | ddPCR primer probe sets (PP sets). *Clcn1* assays were designed using Primer3 software[4, 5], 5′-labeled with either Fam (ex 7a inclusion) or Hex (ex 7a exclusion), and double quenched with internal Zen and 3′ Iowa Black FQ (Zen/IBFQ; IDT). Probes for *ACTA1* mRNA[11] and pre-mRNA[3], and mouse *Dmpk* mRNA[11] were 5′-labeled with Fam and either double quenched with Zen/IBFQ or 3′-labeled with Black Hole Quencher-1 (BHQ-1; Biosearch Technologies). The amplicon size in base pairs (bp) is indicated for each.

PP set	Left primer (5′ - 3′)	Right primer (5′ - 3′)	Probe (5′ - 3′)	Amplicon size (bp)
Clcn1 ex 7a inclusion	GGCGTGGGATGCTACTTTG	ACAGCCCACTGTCAGGATG	CCCCTGATGGAGCAGCCATAC	73
Clcn1 ex 7a exclusion	CTCAGCAAGTTTATGTCCATGTTC	ACAGCCCACTGTCAGGATG	CTGGTGTCTATGAGCAGCCATACT	78
ACTA1 mRNA	GTAGCTACCCGCCCAGAAACT	CCAGGCCGGAGCCATT	ACCACCGCCCTCGTGTGCG	83
ACTA1 pre-mRNA	ctgtccttgcagAAACTAGACAC	CCAGGCCGGAGCCATT	ACCACCGCCCTCGTGTGCG	79
Dmpk mRNA	GACATATGCCAAGATTGTGCACTAC	CACGAATGAGGTCCTGAGCTT	AACACTTGTCGCTGCCGCTGGC	92

Supplementary References

1. Mankodi, A., Logigian, E., Callahan, L., McClain, C., White, R., Henderson, D., Krym, M. & Thornton, C.A. Myotonic dystrophy in transgenic mice expressing an expanded CUG repeat. *Science* **289**, 1769-1773 (2000).
2. Kanadia, R.N., Johnstone, K.A., Mankodi, A., Lungu, C., Thornton, C.A., Esson, D., Timmers, A.M., Hauswirth, W.W. & Swanson, M.S. A muscleblind knockout model for myotonic dystrophy. *Science* **302**, 1978-1980 (2003).
3. Hu, N., Kim, E., Antoury, L., Li, J., Gonzalez-Perez, P., Rutkove, S.B. & Wheeler, T.M. Antisense oligonucleotide and adjuvant exercise therapy reverse fatigue in old mice with myotonic dystrophy. *Mol Ther Nucleic Acids* **23**, 393-405 (2021).
4. Koressaar, T. & Remm, M. Enhancements and modifications of primer design program Primer3. *Bioinformatics* **23**, 1289-1291 (2007).
5. Untergasser, A., Cutcutache, I., Koressaar, T., Ye, J., Faircloth, B.C., Remm, M. & Rozen, S.G. Primer3--new capabilities and interfaces. *Nucleic Acids Res* **40**, e115 (2012).
6. DiFranco, M., Yu, C., Quinonez, M. & Vergara, J.L. Age-dependent chloride channel expression in skeletal muscle fibres of normal and HSA(LR) myotonic mice. *J Physiol* **591**, 1347-1371 (2013).
7. Wheeler, T.M., Lueck, J.D., Swanson, M.S., Dirksen, R.T. & Thornton, C.A. Correction of ClC-1 splicing eliminates chloride channelopathy and myotonia in mouse models of myotonic dystrophy. *J Clin Invest* **117**, 3952-3957 (2007).
8. Lin, X., Miller, J.W., Mankodi, A., Kanadia, R.N., Yuan, Y., Moxley, R.T., Swanson, M.S. & Thornton, C.A. Failure of MBNL1-dependent post-natal splicing transitions in myotonic dystrophy. *Hum Mol Genet* **15**, 2087-2097 (2006).
9. Antoury, L., Hu, N., Balaj, L., Das, S., Georghiou, S., Darras, B., Clark, T., Breakefield, X.O. & Wheeler, T.M. Analysis of extracellular mRNA in human urine reveals splice variant biomarkers of muscular dystrophies. *Nat Commun* **9**, 3906 (2018).
10. Tang, Z.Z., Yarotskyy, V., Wei, L., Sobczak, K., Nakamori, M., Eichinger, K., Moxley, R.T., Dirksen, R.T. & Thornton, C.A. Muscle weakness in myotonic dystrophy associated with misregulated splicing and altered gating of Ca(V)1.1 calcium channel. *Hum Mol Genet* **21**, 1312-1324 (2012).
11. Wheeler, T.M., Leger, A.J., Pandey, S.K., MacLeod, A.R., Nakamori, M., Cheng, S.H., Wentworth, B.M., Bennett, C.F. & Thornton, C.A. Targeting nuclear RNA for in vivo correction of myotonic dystrophy. *Nature* **488**, 111-115 (2012).
