# Supplementary material for: Correction of Clcn1 alternative splicing reverses muscle fiber type transition in mice with myotonic dystrophy
Source: Nat Commun. 2023 Apr 7;14:1956. doi: 10.1038/s41467-023-37619-1 (PMC10082032; doi:10.1038/s41467-023-37619-1)
Supplement: Supplementary file 3 — Description of Additional Supplementary Files [file 41467_2023_37619_MOESM3_ESM.pdf]

Title: Supplementary Movie 1.
Description: Demonstration of myotonia in the LR41;Mbnl1-/- model. Briefly grasping a three month-old LR41;Mbnl1-/- double homozygous mouse at the base of the tail elicits robust myotonia, manifested as hindlimb stiffness lasting for several seconds. By contrast, grasping an age-matched homozygous LR41 littermate at the base of the tail elicits no visible myotonia, although some of these mice feature electrical myotonia detectable by needle electromyography (EMG) examination.

Title: Supplementary Movie 2.
Description: Demonstration of myotonia in the LR20b model. Briefly grasping a three month-old LR20b mouse at the base of the tail elicits relatively mild myotonia, manifested as transient hindlimb stiffness.
