# Supplementary material for: Correction of Clcn1 alternative splicing reverses muscle fiber type transition in mice with myotonic dystrophy
Source: Nat Commun. 2023 Apr 7;14:1956. doi: 10.1038/s41467-023-37619-1 (PMC10082032; doi:10.1038/s41467-023-37619-1)
Supplement: Supplementary file 6 — Reporting Summary [file 41467_2023_37619_MOESM6_ESM.pdf]

nature portfolio

Corresponding author(s): Thurman WHeeler

Last updated by author(s): Feb 9, 2023

Reporting Summary

Nature Portfolio wishes to improve the reproducibility of the work that we publish. This form provides structure for consistency and transparency in reporting. For further information on Nature Portfolio policies, see our Editorial Policies and the Editorial Policy Checklist.

Statistics

For all statistical analyses, confirm that the following items are present in the figure legend, table legend, main text, or Methods section.

n/a	Confirmed	
☐	☒	The exact sample size (*n*) for each experimental group/condition, given as a discrete number and unit of measurement
☐	☒	A statement on whether measurements were taken from distinct samples or whether the same sample was measured repeatedly
☐	☒	The statistical test(s) used AND whether they are one- or two-sided<br>*Only common tests should be described solely by name; describe more complex techniques in the Methods section.*
☐	☒	A description of all covariates tested
☒	☐	A description of any assumptions or corrections, such as tests of normality and adjustment for multiple comparisons
☐	☒	A full description of the statistical parameters including central tendency (e.g. means) or other basic estimates (e.g. regression coefficient) AND variation (e.g. standard deviation) or associated estimates of uncertainty (e.g. confidence intervals)
☐	☒	For null hypothesis testing, the test statistic (e.g. *F*, *t*, *r*) with confidence intervals, effect sizes, degrees of freedom and *P* value noted<br>*Give P values as exact values whenever suitable.*
☒	☐	For Bayesian analysis, information on the choice of priors and Markov chain Monte Carlo settings
☒	☐	For hierarchical and complex designs, identification of the appropriate level for tests and full reporting of outcomes
☐	☒	Estimates of effect sizes (e.g. Cohen's *d*, Pearson's *r*), indicating how they were calculated

Our web collection on statistics for biologists contains articles on many of the points above.

Software and code

Policy information about availability of computer code

Data collection	Image Lab version 5.2.1 (Bio-Rad); Volocity version 6.3.1 (Perkin Elmer)
Data analysis	Image Lab version 5.2.1 (Bio-Rad); Volocity version 6.3.1 (Perkin Elmer); QuantaSoft version 1.7.4 (Bio-Rad); Prism version 9.4.1 (GraphPad)

For manuscripts utilizing custom algorithms or software that are central to the research but not yet described in published literature, software must be made available to editors and reviewers. We strongly encourage code deposition in a community repository (e.g. GitHub). See the Nature Portfolio guidelines for submitting code & software for further information.

Data

Policy information about availability of data

All manuscripts must include a data availability statement. This statement should provide the following information, where applicable:

- Accession codes, unique identifiers, or web links for publicly available datasets
- A description of any restrictions on data availability
- For clinical datasets or third party data, please ensure that the statement adheres to our policy

Source data are provided in this paper.

Human research participants

Policy information about studies involving human research participants and Sex and Gender in Research.

Reporting on sex and gender	Non-applicable
Population characteristics	Non-applicable
Recruitment	Non-applicable
Ethics oversight	Non-applicable

Note that full information on the approval of the study protocol must also be provided in the manuscript.

Field-specific reporting

Please select the one below that is the best fit for your research. If you are not sure, read the appropriate sections before making your selection.

☒ Life sciences ☐ Behavioural & social sciences ☐ Ecological, evolutionary & environmental sciences

For a reference copy of the document with all sections, see nature.com/documents/nr-reporting-summary-flat.pdf

Life sciences study design

All studies must disclose on these points even when the disclosure is negative.

Sample size	The response to Clcn1 ASO treatment in the LR41;Mbnl1-/- mice was unknown. Therefore, we were unable to choose a sample size ahead of time to ensure adequate power to measure pharmacodynamic activity. Instead we estimated sample sizes based on RT-PCR analysis of Clcn1 ASO exon skipping patterns in a prior study in LR20b mice that showed N = 3 was sufficient to reduce exon inclusion by approximately 75% after 3 weeks of treatment (Wheeler, et al., 2007).
Data exclusions	No data were excluded.
Replication	All attempts at replication were successful.
Randomization	Mice were chosen randomly by genotype and stratified for sex to allow an approximately equal number of females and males.
Blinding	Due to the obvious phenotype differences between LR41;Mbnl1-/- and LR41, it was impossible to blind for the activity monitoring studies. The MyHC immunofluorescence data of untreated gastrocnemius muscles were unblinded. For splicing analysis, MyHC immunofluorescence quantification, and fiber diameter measurements of TA muscles, the primary examinter was blinded to ASO treatment assignments and group allocation during data collection. However, the difference between groups was so robust that it effectively identified the invert control oligo treatment groups from the Clcn1 ASO treatment groups prior to unblinding. The ddPCR data were obtained unblinded.

Reporting for specific materials, systems and methods

We require information from authors about some types of materials, experimental systems and methods used in many studies. Here, indicate whether each material, system or method listed is relevant to your study. If you are not sure if a list item applies to your research, read the appropriate section before selecting a response.

Materials & experimental systems

n/a	Involved in the study	
☐	☒	Antibodies
☒	☐	Eukaryotic cell lines
☒	☐	Palaeontology and archaeology
☐	☒	Animals and other organisms
☒	☐	Clinical data
☒	☐	Dual use research of concern

Methods

n/a	Involved in the study	
☒	☐	ChIP-seq
☒	☐	Flow cytometry
☒	☐	MRI-based neuroimaging

Antibodies

Antibodies used	Primary antibodies: anti-MBNL1 rabbit polyclonal antibody (2 µg/ml in PBS; Abcam product number ab45899); anti-MBNL2 (3B4) mouse monoclonal (isotype IgG2b; Santa Cruz Biotechnology, Inc. product number sc-136167); mouse monoclonal BA-F8 (isotype

	IgG2b; myosin heavy chain Type 1), SC-71 (isotype IgG1; myosin heavy chain Type 2A), and BF-F3 (isotype IgM; myosin heavy chain Type 2B) (Developmental Studies Hybridoma Bank, University of Iowa; all deposited by S. Schiaffino, University of Padova); mouse monoclonal 6H1 (isotype IgM; Developmental Studies Hybridoma Bank, University of Iowa; deposited by C. Lucas, University of Sydney); anti-rat CLC-1 IgG rabbit polyclonal antibody (Alpha Diagnostic International product number CLC11-A); Secondary antibodies: goat anti-rabbit Alexa 488 (Invitrogen product number A11034) and goat anti-mouse IgG2b Alexa 546 (Invitrogen product number A-21143); Alexa 647 goat anti-mouse IgG2b (Invitrogen product # A-21242); Alexa 488 goat anti-mouse IgG1 (Invitrogen product # A-21121); Alexa 546 goat anti-mouse IgM (Invitrogen product # A-21045)
Validation	Anti-MBNL1 rabbit polyclonal antibody (2 µg/ml in PBS; Abcam product number ab45899) together with anti-MBNL2 (3B4)<br>Manufacturer information: https://www.abcam.com/mbnl1-antibody-ab45899.html<br>antigen synthetic peptide corresponding to human MBNL1 amino acids 250 - 350; reacts with human, predicted to work with mouse and rat MBNL1; recommended applications immunofluorescence, immunohistochemistry, Western blot.<br>aanti-MBNL2 mouse monoclonal antibody (isotype IgG2b; 4 µg/ml PBS; Santa Cruz Biotechnology, Inc. product number sc-136167)<br>Manufacturer information: https://www.scbt.com/p/mbnl2-antibody-3b4/ antigen recombinant human MBNL2 protein; recommended for detection of MBNL2 of rat, mouse, and human origin recommended applications: immunofluorescence, immunocytochemistry, immunoprecipitation, and Western blot.<br><br>Mouse monoclonal antibody BA-F8 (isotype IgG2b; myosin heavy chain Type 1) at 5 µg/ml PBS<br>DSHB information: https://dshb.biology.uiowa.edu/BA-F8 antigen MyHC 2B, immunogen purified myosin from adult bovine atrium; positive tested species reactivity human, llama, mouse, rat, sheep<br>recommended applications: immunofluorescence, immunohistochemistry, Western blot; antibody registry ID AB_10572253.<br><br>Mouse monoclonal antibody SC-71 (isotype IgG1; myosin heavy chain Type 2A)<br>DSHB information: https://dshb.biology.uiowa.edu/SC-71 antigen myosin 2A, immunogen purified myosin isolated from subcutaneous bovine muscle; positive tested species reactivity bovine, canine, goat, horse, human, mole, mouse, porcine, rabbit, rat, sheep; recommended applications: immunofluorescence, immunohistochemistry, Western blot; antibody registry ID AB_2147165.<br><br>Mouse monoclonal antibody BF-F3 (isotype IgM; myosin heavy chain Type 2B) at 5 µg/ml PBS (Developmental Studies Hybridoma Bank, University of Iowa; all deposited by S. Schiaffino, University of Padova)<br>DSHB information: https://dshb.biology.uiowa.edu/BF-F3 antigen myosin 2B immunogen purified myosin from fetal bovine skeletal muscle; positive tested species reactivity bovine, mouse, porcine, rat, sheep; recommended applications ELISA, immunofluorescence, immunohistochemistry, Western blot; antibody registry ID AB_2266724.<br><br>Mouse monoclonal antibody 6H1 (isotype IgM) at 5 µg/ml PBS (Developmental Studies Hybridoma Bank, University of Iowa; deposited by C. Lucas, University of Sydney)<br>DSHB information: https://dshb.biology.uiowa.edu/6H1 antigen myosin 2X immunogen adult rabbit retractor bulbi crude muscle extract; positive tested species reactivity: feline, guinea pig, human, mouse, rabbit, rat; recommended applications: immunofluorescence, immunohistochemistry, Western blot; antibody registry ID: AB_2314830.<br><br>Affinity purified anti-rat CLC-1 IgG rabbit polyclonal antibody (20 µg/ml in PBS; Alpha Diagnostic International product number CLC11-A); Manufacturer information: https://4adi.com/4adi/anti-rat-chloride-channel-1-clc1-igg-1-aff-pure-10744-p.html antigen 18-amino acid peptide of rat ClC-1 100% conserved in mouse, dog, rat, and human; species cross-reactivity not studied; suggested applications immunofluorescence, immunohistochemistry, and Western blot.

Animals and other research organisms

Policy information about studies involving animals; ARRIVE guidelines recommended for reporting animal research, and Sex and Gender in Research

Laboratory animals	We used the Human Skeletal Actin Long Repeat (HSALR) line 20b (LR20b) and line 41 (LR41) transgenic mouse model, and the muscleblind-like 1 knockout mouse model. Wild type FVB mice served as controls. All mice were maintained on the FVB background. Age ranged from 7 weeks to 18 months. All mice were fed standard chow ad libitum and housed in groups of up to five. Lighting was time controlled on a standard 12:12 light/dark cycle. Temperature and humidity were stable and consistent at 20 - 25 degrees Celsius and 30 - 70%, respectively.
Wild animals	We used no wild animals in this study.
Reporting on sex	Experimental mice were stratified by sex to allow an approximately equal number of females and males.
Field-collected samples	We used no field-collected samples in this study.
Ethics oversight	The MGH IACUC approved all mouse studies.

Note that full information on the approval of the study protocol must also be provided in the manuscript.
